# Supplementary material for: Analysis of PPARγ Signaling Activity in Psoriasis
Source: Int J Mol Sci. 2021 Aug 10;22(16):8603. doi: 10.3390/ijms22168603 (PMC8395241; doi:10.3390/ijms22168603)
Supplement: Supplementary file 1 [file ijms-22-08603-s001.zip › Supplemental materials_Analysis of PPARg signaling activity in psoriasis/Pathway models/Models images and html files/PPARG expressed in cells assotiated with psoriasis/226605.html]

Th1 cell


# CellType Th1 cell

|  |  |
| --- | --- |
| URN | urn:agi-ncimcelltype:C0242632 |
| Total Entities | 49 |
| Connectivity | 4162 |
| Name | Th1 cell |

---

|  |  |
| --- | --- |
| ChildConcepts | T helper cell function |
|  | T-helper 1 cell differentiation |
|  | T-helper lymphocyte infiltration |
|  | Th1 cell population |
|  | Th1 cell proliferation |
|  | Th1 cell repertoire |
|  | Th1 cell development |
|  | Th1 cell polarity |
|  | Th1 cell aggregation |
|  | Th1 cell formation |
|  | Th1 cell fate commitment |
|  | Th1 cell phenotype |
|  | Th1 cell ratio |
|  | Th1 cell death |
|  | Th1 cell survival |
|  | Th1 cell priming |
|  | Th1 cell apoptosis |
|  | Th1 cell migration |
|  | Th1 cell clonal anergy |
|  | Th1 cell cytotoxicity |
|  | Th1 cell rolling |
|  | Th1 cell transendothelial migration |
|  | Th1 cell chemotaxis |
|  | Th1 cell mediated cytotoxicity |
|  | Th1 cell fate |
|  | Th1 cell proliferative response |
|  | Th1 cell contact |
|  | Th1 cell diapedesis |
|  | Th1 cell adhesion |
|  | Th1 cell transepithelial migration |
|  | Th1 cell growth |
|  | Th1 cell sequestration |
|  | Th1 cell extravasation |
|  | Th1 cell homing |
|  | Th1 cell viability |
|  | Th1 cell localization |
|  | Th1 cell autophagy |
|  | Th1 cell homeostasis |
|  | Th1 cell fate determination |
|  | Th1 cell selection |
|  | Th1 cell compartmentalization |
|  | Th1 cell tethering |
|  | Th1 cell motion |
|  | Th1 cell distribution |
|  | Th1 cell interaction |
|  | Th1 cell developmental process |
|  | Th1 cell damage |
|  | Th1 cell invasion |
|  | Th1 cell disruption |

---

|  |  |
| --- | --- |
| Pathway | STAT3 Facilitates the Function of Treg Cells and Cancer Progression |
|  | PPARG expressed in cells assotiated with psoriasis |

---

|  |  |
| --- | --- |
| MedScan ID | 10003434 |

---

|  |  |
| --- | --- |
| Alias | Th1 cell |
|  | type 1 helper cells |
|  | T helper Th1 cells |
|  | T helper type 1 |
|  | T helper 1 Th1 cells |
|  | type 1 T helper |
|  | T helper 1 cell |
|  | T helper 1 cells |
|  | T H 1 cell |
|  | T H 1 cells |
|  | T helper 1 Th1 cell |
|  | helper cell type 1 |
|  | type 1 T helper cells |
|  | type 1 helpers |
|  | T helper Th1 cell |
|  | Th1 cells |
|  | type 1 helper T cell |
|  | type 1 helper cell |
|  | T-cell helper type 1 |
|  | T helpers type 1 |
|  | type 1 T helper cell |
|  | type 1 T helpers |
|  | type 1 helper T cells |
|  | T-helper cell type 1 |

---

|  |  |
| --- | --- |
| NCIm ID | C0242632 |

---
